# Influence of Particle Size, Defect Density and Salts on the Dissolution and Early Hydration of a Model System (C_3_A + Quartz)

**DOI:** 10.3390/ma18153560

**Published:** 2025-07-29

**Authors:** Shaoxiong Ye, Pan Feng

**Affiliations:** 1 College of Civil Engineering, Huaqiao University, Xiamen 361021, China; 2 Jiangsu Key Laboratory of Construction Materials, School of Materials Science and Engineering, Southeast University, Nanjing 211189, China; 3 State Key Laboratory of High Performance Civil Engineering Materials, Nanjing 210008, China

**Keywords:** model cement, dissolution and early hydration, defect density, particle size, salt effect

## Abstract

Understanding the hydration behavior of cementitious materials is crucial as it governs the setting, strength development and long-term durability of concrete. This study provides fundamental insights into these processes by investigating the early hydration of tricalcium aluminate (C_3_A) with quartz as a novel model system for multiple clinker phases. Employing a multi-technique approach combining conductivity, calorimetry and microscopy, we systematically examine the concurrent effects of product layer formation, C_3_A’s particle size and defect density, and salts on dissolution kinetics and early-stage reaction pathways. Results indicate that product layer formation shifted C_3_A’s rapid dissolution toward diffusion-controlled regimes. Reduced particle size and increased defect density accelerated the dissolution and hydration kinetics. Sulfates and chlorides differentially altered reaction pathways, with preferential sulfate reactivity driving ettringite formation. These mechanistic insights advance fundamental understanding of the hydration behavior of cementitious material.

## 1. Introduction

Portland cement, with annual global production about 4 billion tonnes, serves as the fundamental building block of modern infrastructure. Its complex hydration process, a coupled dissolution–precipitation reaction, directly dictates the development of microstructure, mechanical properties and durability in concrete [1,2,3]. Despite two centuries of empirical optimization, mechanistic understanding of the initial hydration reactions remains critically fragmented, particularly regarding fundamental processes when multiple clinker phases react concurrently [4,5]. Emerging studies [6,7,8] have revealed that the dissolution and nucleation events in the initial stages determine the kinetic pathways that continue throughout subsequent hydration processes. Herein lies the pivotal role of tricalcium aluminate (C_3_A), the most reactive clinker phase, where its rapid dissolution kinetics control the initial solution supersaturation and thereby dictate hydrate assembly pathways [9,10,11]. Consequently, decoding C_3_A’s early hydration mechanisms represents an urgent scientific priority.

The pronounced reactivity of C_3_A fundamentally arises from its distinctive crystal structure (characterized by cubic or orthorhombic arrangements of Ca-O polyhedra with Al atoms in tetrahedral coordination), which generates metastable bonding networks inherently prone to hydrolysis [12,13,14]. This structural instability drives rapid heterogeneous dissolution upon aqueous contact, initiating localized etch pit formation within 0.1 s [7]. To mitigate the consequent risk of flash setting, soluble sulfates are introduced to modulate C_3_A’s hydration kinetics [15,16]. Specifically, C_3_A reacts with sulfates to form ettringite (AFt) within minutes, followed by a characteristic induction period before subsequent reaction resurgence converts AFt to monosulfate (AFm) [15,17]. Despite C_3_A’s critical role in governing early reaction kinetics, the mechanistic basis of gypsum’s retardation effect on its hydration remains contested [9,10], and its dissolution kinetics remain inadequately quantified under realistic conditions [7,16].

In reality, dissolution initiates preferentially at localized defect sites, such as dislocations, vacancies and grain boundaries, as extensively documented in geochemistry [18,19]. However, this fundamental principle remains critically underexplored in cement science, particularly regarding C_3_A’s hydration. Conventional solid-state synthesized C_3_A develops crystallographic defects during cooling that profoundly influence dissolution kinetics: slow cooling permits dislocation annihilation, yielding low-defect-density crystals, whereas quenching traps dislocations and vacancies, creating high-energy dissolution initiation sites [20,21]. Despite extensive evidence of defect-mediated reactivity in mineral systems [22,23,24], prevailing cement hydration models assume C_3_A’s surface homogeneity, neglecting anisotropic dissolution behavior [8,11]. This oversimplification consequently impedes prediction of early-stage kinetics governed by defect density, representing a significant limitation given C_3_A’s pivotal role in setting control. Crucially, the quantitative impact of annealing protocols on C_3_A’s dissolution and early hydration remains unestablished.

While commercial Portland cement’s four primary clinker phases hydrate synergistically, this complexity obscures fundamental reaction mechanisms. Cement hydration emerges not merely as the sum of individual phase reactions, but through competitive dissolution dynamics and complex interactions [25,26,27,28]. Crucially, sulfate optimization governs the early kinetic pathways: at industrially relevant alite/C_3_A ratios, gypsum’s dosage determines reaction sequence. In properly sulfated systems, calcium sulfate depletion and renewed hydration of C_3_A occur after the main alite reaction. Conversely, undersulfated conditions trigger premature C_3_A’s reaction, forming AFm before the main alite reaction [25,26,29]. This sulfate-mediated competition extends to aluminate phases in clinker, where C_3_A preferentially consumes sulfate over tetracalcium aluminoferrite [30,31]. Evidence confirms near-complete AFt formation from C_3_A alongside residual unreacted tetracalcium aluminoferrite [32], demonstrating that co-dissolution behavior in multiphase systems defies prediction from isolated phase studies. Such emergent phenomena fundamentally challenge reductionist approaches to hydration modeling, so model systems isolating C_3_A provide essential scientific reductionism without sacrificing chemical relevance. Pairing C_3_A with inert quartz enables precise interrogation of how intrinsic particle properties (defects and particle size) and extrinsic solution factors (saturation and salt types) modulate ion release kinetics without the interference of alite’s dissolution–nucleation coupled reaction, as demonstrated by Axthammer et al. [33] and Maier et al. [34]. Quartz not only provides a granular packing similar to cement while maintaining chemical inertness, but also preserves aluminate-dominated solution chemistry. This allows kinetic phenomena to be attributed unambiguously to C_3_A’s reactivity, thereby isolating the phase interactions that are obscured in full systems.

Given this context, this study addresses the critical knowledge gap in C_3_A’s hydration by systematically manipulating several underexplored variables (product layer formation, particle size, crystallographic defect density and dissolved salts including sulfates and chlorides) through a controlled C_3_A–quartz model system. A multi-method approach was employed to establish mechanistic causality: conductivity quantifies defect-mediated dissolution kinetics in real-time, isothermal calorimetry tracks early hydration pathways and microscopy resolves evolving microstructural evolution. This integrated methodology provides comprehensive insights into early hydration mechanisms, which are essential for designing advanced cementitious materials.

## 2. Materials and Methods

Material Synthesis: C_3_A with 96.73% purity was prepared via solid-state reaction following [35]. The resulting C_3_A was ground and sieved to produce three batches with different particle size distributions: one labeled coarse (C-C_3_A, D_50_ = 34.521 μm, specific surface area = 0.387 m^2^·g^−1^), one labeled median (M-C_3_A, D_50_ = 20.811 μm) and one labeled fine (F-C_3_A, D_50_ = 9.083 μm, specific surface area = 0.533 m^2^·g^−1^). The particle size distributions were quantified using a particle size analyzer (PSA 1190 LD, Anton Paar, Graz, Austria), while the specific surface areas were determined using an Autosorb iQ Station 2 (Quantachrome, Boynton Beach, FL USA) under a nitrogen atmosphere. To engineer reduced defect density, some of the fine batch subsequently underwent thermal annealing at 600 °C for 6 h. Analytical-grade quartz (Q, D_50_ = 12.6 μm, supplied by Sinopharm Chemical Reagent Co., Ltd., Shanghai, China) was blended with C_3_A at a 92:8 mass ratio [25] to approximate commercial cement composition using a Turbula T2F mixer (WAB Willy A. Bachofen AG, Muttenz, Switzerland), forming the C_3_A–quartz model system.

Solution Design: Analytical-grade reagents from Sinopharm Chemical Reagent Co., Ltd., gypsum (CaSO_4_·2H_2_O), sodium chloride (NaCl) and calcium chloride (CaCl_2_), were dissolved in ultrapure water (resistivity >18 MΩ·cm at 25 °C) generated by a Smart-S15 water purification system (HHitech, Shanghai, China) to prepare aqueous solutions of varying concentrations. Due to gypsum’s limited solubility, CaSO_4_ solutions had concentrations of 1 mmol·L^−1^ or 10 mmol·L^−1^. NaCl solutions were prepared at 2 mmol·L^−1^, 10 mmol·L^−1^, 20 mmol·L^−1^ or 50 mmol·L^−1^, while CaCl_2_ solutions were prepared at 1 mmol·L^−1^, 5 mmol·L^−1^, 10 mmol·L^−1^ or 25 mmol·L^−1^. Additionally, analytical-grade sodium aluminate (NaAlO_2_, supplied by Aladdin Scientific Corp., Shanghai, China) was used to prepare aqueous solutions for validating concentration-dependent conductivity relationships.

### 2.1. Dissolution and Early Hydration Kinetic Tracking

Hydration reactions were monitored via synchronized conductivity–pH measurements and isothermal calorimetry.

A conductivity–pH meter (SevenExcellence, Mettler Toledo, Greifensee, Switzerland) continuously monitored both conductivity and pH in C_3_A–quartz suspensions to track hydration kinetics. The measurements were conducted in a glass beaker maintained at 20 °C by a water bath kettle and prior to each test, the instrument was calibrated by buffer solutions. Specifically, ultrapure water or an aqueous solution of sulfate or chloride was first added to the beaker and equilibrated at 20 ± 0.5 °C. Then, pre-weighed C_3_A–quartz mixture was introduced, after which the instrument started automated data collection at a time step of 5 s upon solid–liquid contact. A magnetic stirrer maintained homogeneity of the suspension throughout testing, while a plastic film was used to minimize the effects of evaporation and carbonation.

The hydration heat flow of quartz–C_3_A mixtures was monitored using an eight-channel isothermal calorimeter (TAM Air, TA Instruments, New Castle, DE, USA) at 20 °C. Pre-weighed mixtures were loaded into glass ampoules, while ultrapure water was loaded into calibrated syringes. After thermal equilibration within the calorimeter, hydration was initiated by injecting water into the ampoules. Heat flow measurements were commenced immediately upon mixing and continued for 1 h.

To ensure repeatability, a minimum of three replicate experiments were conducted for each condition.

### 2.2. Characterization of Hydrated Samples

Some hydrated C_3_A powders were collected for morphology and component analyses after prescribed reaction times. Reactions were arrested by a liquid nitrogen freeze-drying method to preserve their microstructure, followed by product layer thickness quantification, morphological analysis and phase identification.

Optical microscopy (OM) and scanning electron microscopy (SEM) were employed to characterize the morphology of hydrated C_3_A powders. For OM analysis, product layer thickness was quantified using an optical microscope (BM2100POL, Yongxin Optics, Ningbo, China). Dried specimens were epoxy-impregnated, sequentially ground, and polished to expose transverse sections prior to measurement. At a magnification of 600, randomly selected C_3_A particles were imaged via CCD camera for statistical thickness quantification. For SEM analysis, an FEI Quanta 3D microscope (FEI, Hillsboro, OR, USA) operating at 20 kV in secondary electron mode with integrated EDX was utilized to examine reaction-stage morphologies. All samples were sputter-coated with ~50 nm platinum prior to image capturing.

X-ray diffraction analysis (XRD) was performed on a Bruker D8 Advance diffractometer (Bruker, Karlsruhe, Germany) using Cu Kα radiation at 40 kV and 40 mA. Diffraction patterns were acquired over a 2θ range of 7° to 25° with a step size of 0.02° and scan rate of 0.3 s/step.

## 3. Results and Discussion

### 3.1. Role of Product Layer Formation on Early Reaction

**Figure 1:**
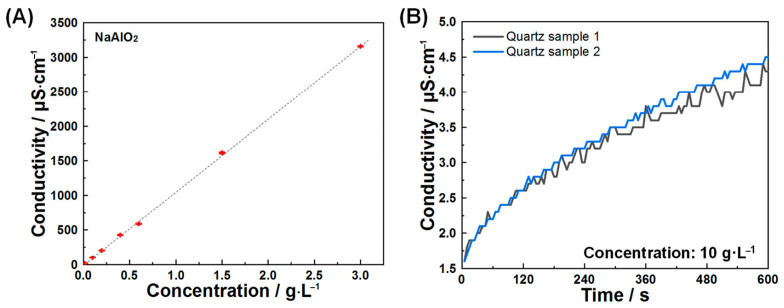
(**A**) Conductivities of solutions as a function of NaAlO_2_ concentrations and (**B**) temporal evolution of conductivity in quartz suspensions during early-stage dissolution.

Conductivity testing is a key method for characterizing macroscopic mineral dissolution, as it relies on the linear proportionality between conductivity and total ions released from dissolution [36]. In the case of sodium aluminate (Figure 1A), conductivity increased linearly with rising concentrations of aluminate and sodium ions. This established relationship enables quantitative determination of C_3_A’s dissolution rates.

To isolate dissolution behavior specifically attributable to C_3_A, quartz was used as an inert reference material. Quartz dissolution induced negligible conductivity variations (Figure 1B) while exhibiting no significant interactions with ions released during C_3_A dissolution [37]. These properties collectively enable effective simulation of steric hindrance effects exerted by silicate phases on C_3_A’s dissolution in cementitious systems.

**Figure 2:**
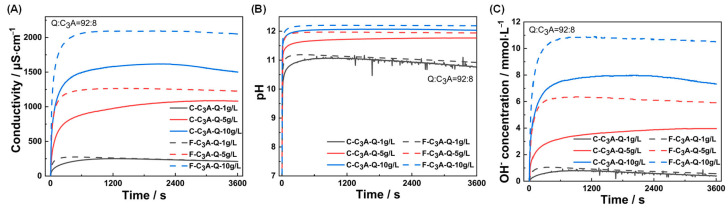
Temporal evolutions of (**A**) conductivity, (**B**) pH value and (**C**) OH- concentration of suspensions with solid concentrations of 1 g·L^−1^, 5 g·L^1^ or 10 g·L^−1^.

Figure 2 depicts the evolution of conductivity, pH and hydroxide ion concentration in C_3_A–quartz suspension during dissolution. As shown in Figure 2A, all suspensions exhibited a linear conductivity increase stage within the first tens of seconds, followed by a stabilization period. Higher mixture concentrations yielded greater conductivity values, while finer C_3_A accelerated conductivity growth rates at identical concentrations. The pH variation pattern (Figure 2B) paralleled conductivity trends. Hydroxide ion concentration profiles (Figure 2C) were further derived from pH values using their temperature-dependent correlation. Based on hydroxide ion concentration change rates during the 0–30 s linear phase, C_3_A’s specific surface areas, and the dissolution kinetics equation [38], the following equation was generated:

(1) $${\mathrm{Ca}}_{3}{\mathrm{Al}}_{2}O_{6}{+6H}_{2}O=3{\mathrm{Ca}}^{2+}{+2\mathrm{Al}(\mathrm{OH})}_{4}^{-}+{4\mathrm{OH}}^{-}$$

From this, the macroscopic dissolution rates were calculated as 0.095 mmol·m^−2^·s^−1^ for C-C_3_A and 0.138 mmol·m^−2^·s^−1^ for F-C_3_A. These powder dissolution rates share the same order of magnitude with Stage III dissolution rates of bulk C_3_A measured by DHM (reported in [7]), suggesting identical reaction-controlling mechanisms.

**Figure 3:**
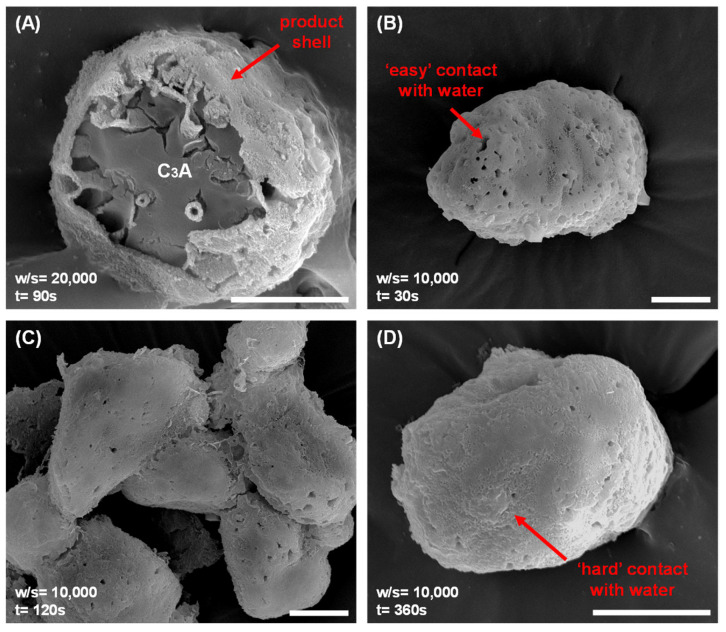
Morphological evolution of C_3_A powders during reaction, preserved by liquid nitrogen freeze-drying: after (**A**) 90 s reaction in a suspension with a w/s of 20,000, and after (**B**) 30 s, (**C**) 120 s and (**D**) 3600 s reaction in a suspension with a w/s of 10,000. Scale bar, 10 μm.

Morphological evolution during C_3_A’s dissolution provides critical insights into reaction-controlling mechanisms. To preserve the morphology of C_3_A at specific dissolution times in water, samples were rapidly frozen using liquid nitrogen and freeze-dried, as shown in Figure 3. Analysis of these preserved samples revealed that surface product layers formed very rapidly on the C_3_A particles. Critically, Figure 3A demonstrates that these layers were already present after just 90 s, even at a very high water-to-solid ratio (w/s = 20,000). Further, lower w/s ratios (such as 10,000, shown in Figure 3B–D) accelerated this layer formation. As the reaction time increased from 30 s to 3600 s at w/s = 10,000, the product layers became progressively denser. This densification indicates a reduction in the direct contact channels between unreacted C_3_A and the surrounding water. Combined with supporting conductivity and pH measurements, these morphological observations establish that the formation of these surface product layers is the primary reason for the significant decrease observed in C_3_A’s dissolution rates.

**Figure 4:**
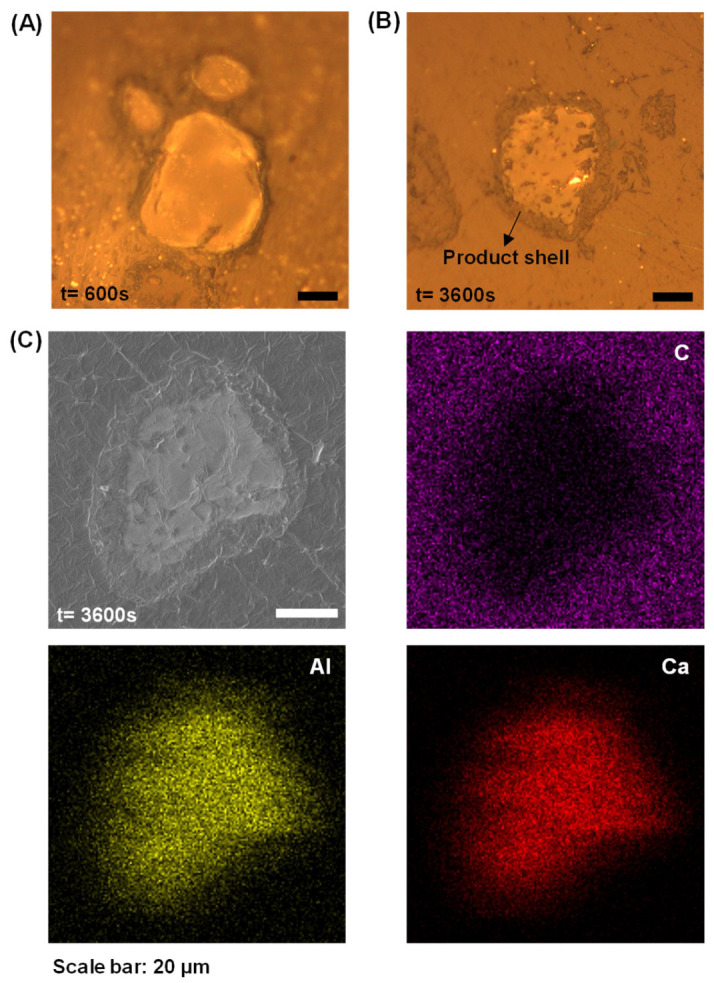
Cross-sectional analysis of hydrated C_3_A revealing layer growth and chemical evolution: optical images of cross-sections of C_3_A powders after (**A**) 600 s and (**B**) 3600 s reaction, and (**C**) the morphology and element mapping of a cross-section of C_3_A powder after 3600 s reaction.

Furthermore, previous conductivity/pH measurements indicate C_3_A’s dissolution quickly entered a stage where conductivity/pH values were essentially constant. This phenomenon could be attributed to either the essentially stopped reaction of C_3_A or the establishment of dissolution–precipitation equilibrium [1]. As presented in Figure 4A,B, these optical micrographs reveal progressively thickening dark rims surrounding reacted C_3_A particles, with rim darkness intensifying over time. These rims represent product layers exhibiting lower optical reflectance than unhydrated C_3_A cores. SEM-EDX mapping (Figure 4C) corroborates this interpretation, showing significant depletion of aluminum and calcium at particle peripheries, definitive evidence of product layer formation.

**Figure 5:**
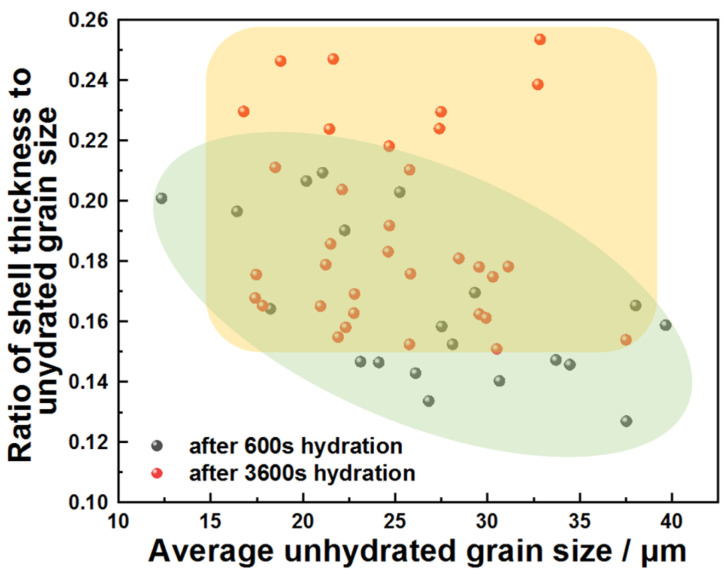
Distribution of the ratio of product shell thickness to unhydrated grain size of C_3_A powders with different average unhydrated grain sizes after 600 s or 3600 s reaction in water.

Growth kinetics of the product layer serve as a critical indicator for mechanism discrimination: continuous thickening during the stabilization stage confirmed dissolution–precipitation equilibrium, whereas a constant thickness indicated that the reaction had ceased. Figure 5 quantifies the thickness ratio of product layers to unreacted cores for different C_3_A particle sizes at 600 s and 3600 s, where higher ratios indicate greater reaction extent. The statistically distinct clustering of ratios (3600 s > 600 s) demonstrated progressive hydration between 600 s and 3600 s.

Integrating the aforementioned findings, the early-stage dissolution process and controlling mechanisms of C_3_A’s reaction in pure water are summarized as follows: C_3_A undergoes initial rapid dissolution releasing substantial ions, followed by swift attainment of solution supersaturation that triggers nucleation and growth of products on C_3_A’s surface, consistent with the literature [7]. The product layer subsequently acts as a transport barrier between unreacted C_3_A and water, and this barrier effect shifts dissolution kinetics toward diffusion-controlled regimes.

### 3.2. Intrinsic Factors Affecting the Early Reaction

**Figure 6:**
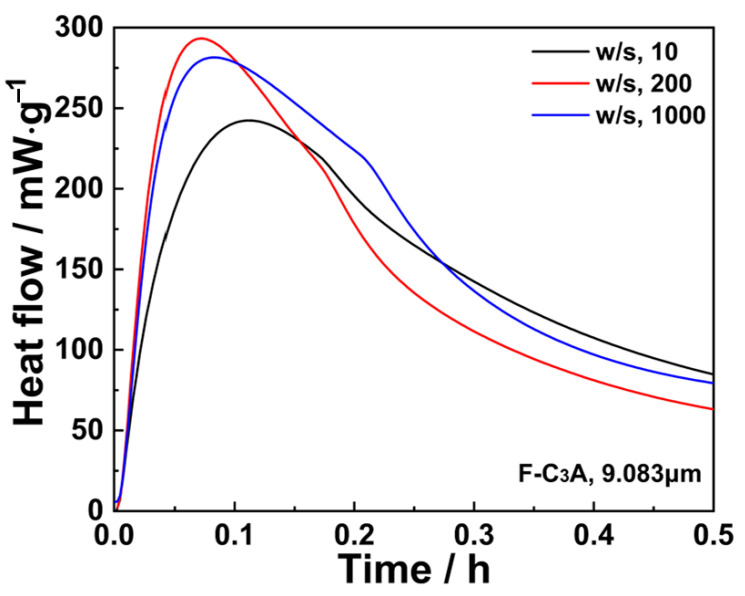
Effect of w/s ratio on the heat flow of C_3_A within the first 0.5 h.

Isothermal calorimetry was employed to systematically evaluate the impact of w/s on the exothermic behavior of C_3_A’s hydration. As demonstrated in Figure 6, elevating w/s from 10 to 200 markedly accelerated early-stage hydration kinetics, which is commonly observed in cement chemistry [39]. This acceleration plateaued when w/s was further increased to 1000, exhibiting minimal reaction rate variation due to comparable solution saturation levels under these conditions. Consequently, calorimetric measurements beyond w/s = 1000 were precluded in this study by instrument capacity limitations.

**Figure 7:**
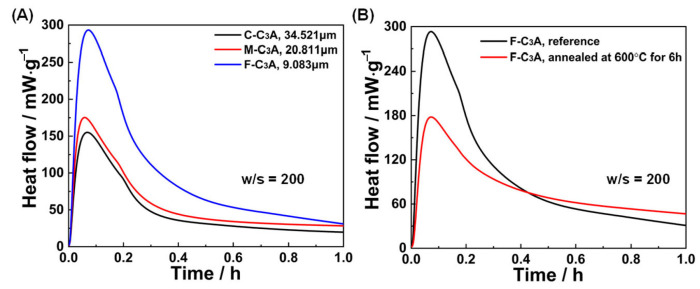
Effects of (**A**) particle size and (**B**) defect density on the first 1 h hydration of C_3_A.

The reactivity of particulate systems usually decreases as particle size increases due to the increased specific surface area. This fundamental principle is validated in Figure 7A, where finer C_3_A particles exhibited accelerated hydration kinetics during the first hour, compared to coarser counterparts. Beyond particle size, atomic-scale defect density also emerged as a governing parameter for hydration kinetics [40]. Thermal annealing at 600 °C for 6 h (Figure 7B) progressively eliminated crystal defects in C_3_A, consequently suppressing heat release rates through reduced reaction site availability.

### 3.3. Sulfates and Chlorides Affected the Early Reaction

**Figure 8:**
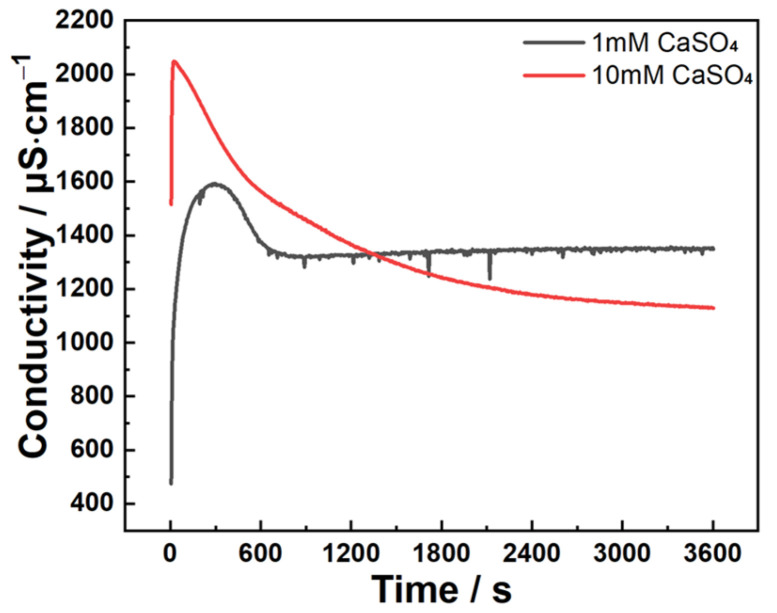
Conductivity evolutions of C_3_A–quartz mixtures when reacting in CaSO_4_ solutions.

Figure 8 presents conductivity evolution profiles of C_3_A powder reacting in 1 mmol·L^−1^ and 10 mmol·L^−1^ CaSO_4_ solutions. Both curves exhibit an initial ascent to peak values followed by gradual decline. Notably, unlike the 10 mmol·L^−1^ system, conductivity in the 1 mmol·L^−1^ solution transitions to a plateau stage after approximately 810 s of descent.

**Figure 9:**
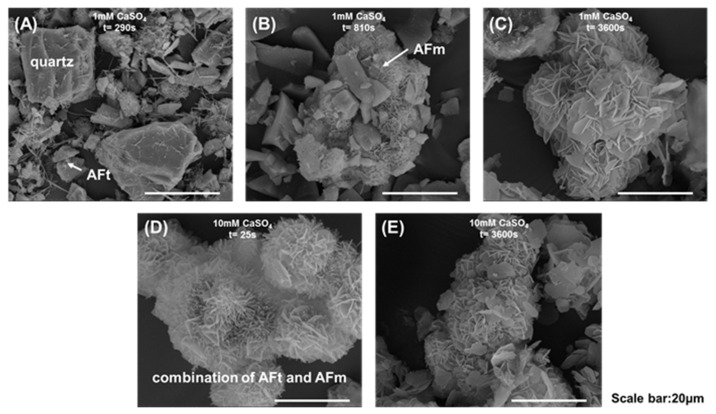
Morphologies of C_3_A–quartz mixtures after reacting in a 1 mmol·L^−1^ CaSO_4_ solution for (**A**) 290 s, (**B**) 810 s and (**C**) 3600 s, and after reacting in a 10 mmol·L^−1^ CaSO_4_ solution for (**D**) 25 s and (**E**) 3600 s.

**Figure 10:**
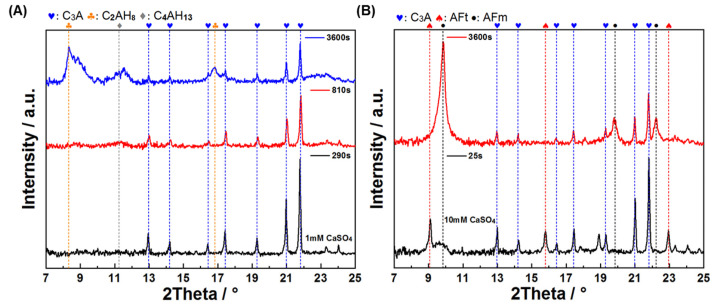
XRD patterns of C_3_A after reacting (**A**) in a 1 mmol·L^−1^ CaSO_4_ solution and (**B**) in a 10 mmol·L^−1^ CaSO_4_ solution for different times.

Morphological and phase composition analyses were conducted at characteristic points of these curves (Figure 9 and Figure 10). For XRD characterization, pure C_3_A samples were used instead of C_3_A–quartz mixtures to avoid analytical interference, as dominant quartz diffraction peaks in blended systems would obscure hydration product reflections. Figure 9 presents the morphological progression of C_3_A’s hydration in CaSO_4_ solutions: in the 1 mmol·L^−1^ system, needle-shaped products evolved into foil-dominated structures by 810 s, culminating in exclusive foil morphology at 3600 s; conversely, the 10 mmol·L^−1^ system exhibited primarily needle-shaped morphologies with minor foils at 25 s before transitioning to pure foils by 3600 s. XRD analysis (Figure 10) revealed distinct phase pathways: for 1 mmol·L^−1^, C_3_A’s peaks progressed to broad humps at 8~9° and 11.2° 2θ (810 s) [41], later crystallizing into sharp peaks for calcium aluminate hydrates C_2_AH_8_ and C_4_AH_13_ (3600 s); in 10 mmol·L^−1^, early AFt/AFm coexistence (25 s) converted entirely to AFm by 3600 s. Crucially, SEM and XRD correlation demonstrated that identical foil morphologies hosted divergent phases.

These findings decode the conductivity curves: in 1 mmol·L^−1^, the initial ascent corresponds to AFt formation, the peak-to-plateau transition reflects AFt converting to AFm alongside C_2_AH_8_/C_4_AH_13_ formation (enabled by low SO_4_^2−^), with later dominance of these phases; in 10 mmol·L^−1^, the initial ascent similarly denotes AFt growth, but the descent phase solely represents AFt consumption and AFm formation due to abundant sulfate ions.

**Figure 11:**
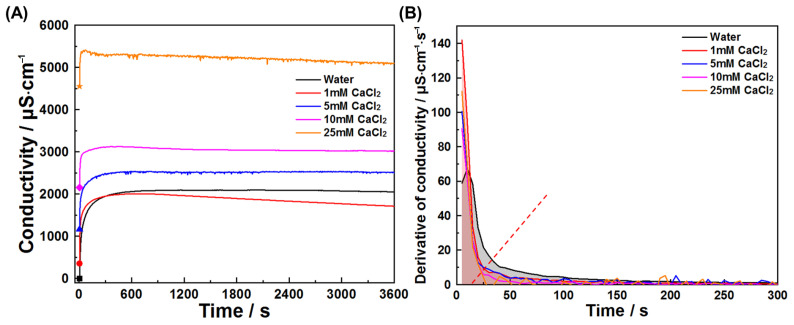
Reaction kinetics of C_3_A–quartz mixtures in CaCl_2_ solutions: (**A**) conductivity evolutions and (**B**) derivative analysis of conductivity evolutions.

**Figure 12:**
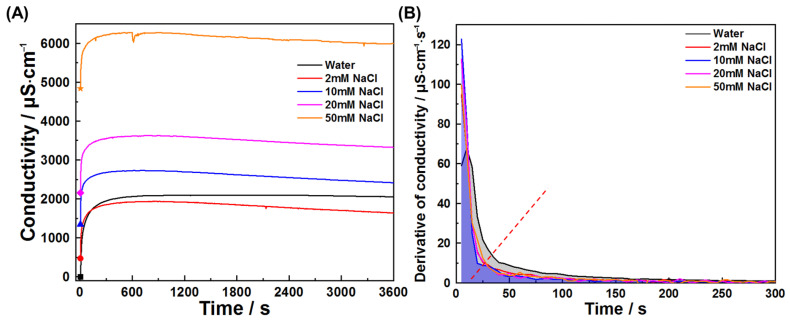
Reaction kinetics of C_3_A–quartz mixtures in NaCl solutions: (**A**) conductivity evolutions and (**B**) derivative analysis of conductivity evolutions.

This study also investigated early hydration of C_3_A in chloride solutions and chloride–sulfate composite systems. Figure 11 and Figure 12 present conductivity profiles and their derivatives for C_3_A–quartz mixtures in CaCl_2_ and NaCl solutions, respectively. Figure 11A reveals similar curve shapes across all solutions, indicating consistent reaction pathways: initial rapid dissolution followed by product nucleation and growth. The pre-inflection conductivity increment decreases with rising CaCl_2_ concentration, signifying reduced reaction extent during product formation. This trend is further elucidated in Figure 11B, where increasing CaCl_2_ concentration progressively reduces derivative curve envelope areas and advances inflection points. Under the assumption that ion consumption by product growth negligibly interferes with dissolution during the initial 20 s, derivative values qualitatively reflect CaCl_2_’s influence on dissolution kinetics, where higher values denote faster dissolution. C_3_A’s dissolution was enhanced in all four CaCl_2_ concentrations tested, though not monotonically with concentration. Similar phenomena are observed for NaCl solutions (Figure 12).

**Figure 13:**
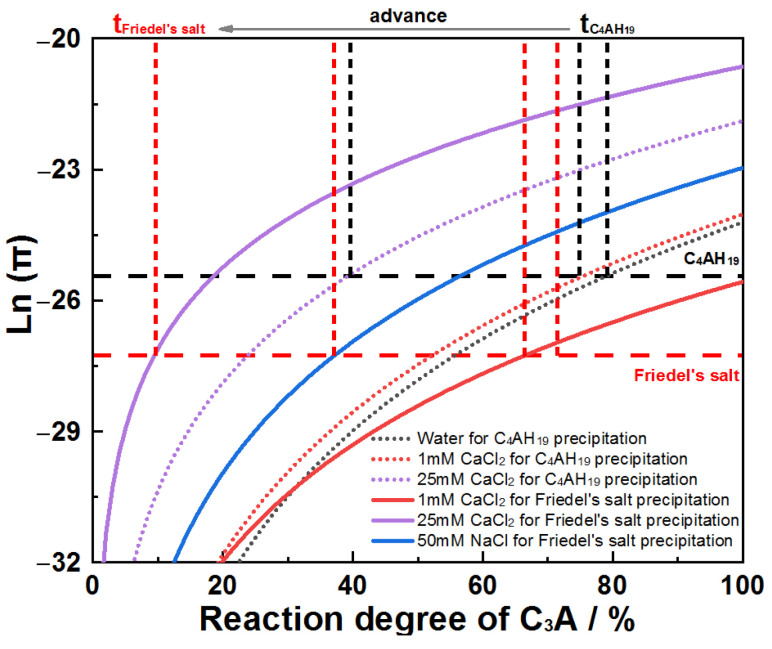
Evolution of Ln(π) as a function of C_3_A reaction degree when hydrating in water and chloride solutions.

The earlier conductivity inflection points observed can be thermodynamically rationalized. Assuming ionic activities’ approximate concentrations at w/s = 100, ion activity products for Friedel’s salt and C_4_AH_19_ (noting that C_4_AH_13_ derives from C_4_AH_19_’s dehydration) were plotted against C_3_A reaction extent across the systems (Figure 13). For all chloride solutions, the reaction extent required to reach Friedel’s salt solubility product (K_sp_) is lower than for C_4_AH_19_, indicating preferential Friedel’s salt formation. This thermodynamic preference explains the advanced inflection points. Notably, the reaction extents at K_sp_ attainment in Figure 13 exceed experimental values due to localized solution supersaturation near particle surfaces, a consequence of diffusion limitations. However, this disparity does not invalidate the conclusion regarding Friedel’s salt prioritization.

**Figure 14:**
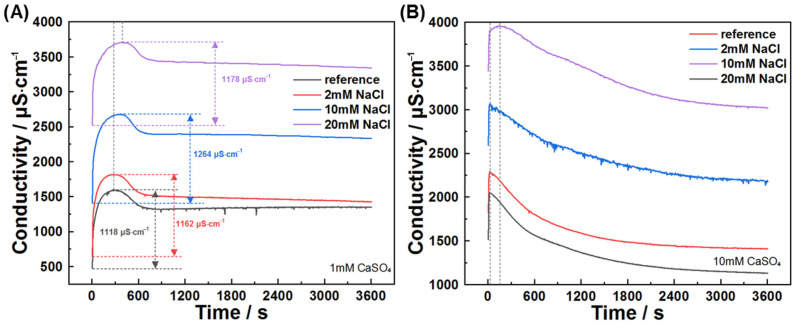
NaCl concentration effect on the conductivity evolution of C_3_A–quartz suspension when hydrating in solutions with (**A**) 1 mmol·L^−1^ CaSO_4_ or (**B**) 10 mmol·L^−1^ CaSO_4_.

**Figure 15:**
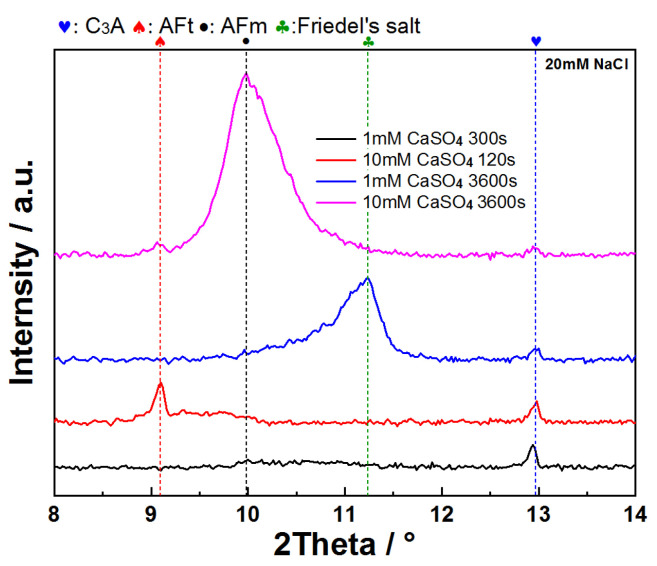
XRD patterns of C_3_A after reacting in CaSO_4_–NaCl solutions for different times.

When C_3_A is dissolved in mixed sulfate–chloride solutions, the antagonistic effects of these ions on dissolution kinetics are superseded by sulfate dominance, as evidenced by suspension conductivity measurements (Figure 14). The conductivity profiles of C_3_A–quartz mixtures in CaSO_4_–NaCl mixtures consistently mirror those in pure CaSO_4_ solutions. Notably, the pre-inflection conductivity increment rises from 1118 μS·cm^−1^ in reference solutions to higher values with NaCl addition, indicating partial counteraction of sulfate’s inhibitory effect that enhances ionic accumulation. XRD analysis (Figure 15) reveals C_3_A’s preferential formation of sulfur-containing hydration products. Only at low sulfate concentrations does Friedel’s salt emerge and evolve into the dominant phase.

**Figure 16:**
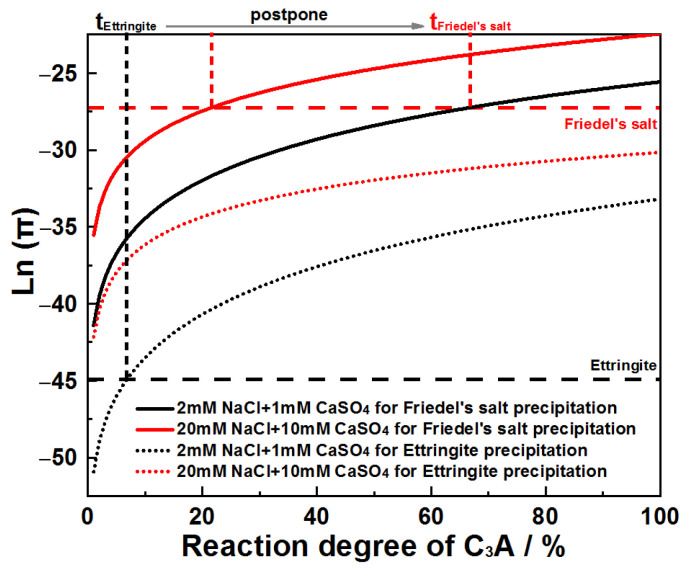
Evolution of Ln(π) as a function of C_3_A reaction degree when hydrating in CaSO_4_–NaCl solutions.

Ion activity products for Friedel’s salt and AFt were plotted against C_3_A’s reaction extent across systems, as presented in Figure 16. Analysis reveals that in CaSO_4_–NaCl mixed solutions, the reaction extent required to reach AFt saturation consistently registers as the lowest value when compared to Friedel’s salt. This thermodynamic priority confirms C_3_A’s preferential formation of AFt.

## 4. Conclusions

This study sheds light on dissolution and early hydration mechanisms in a model C_3_A–quartz system. Through an integrated methodology combining real-time conductivity–pH measurement, calorimetry and cryo-preserved microscopy, we deciphered asymmetric modulation pathways governing early-stage reactions. The characteristic conductivity profiles revealed a critical kinetic transition involving an initial rapid conductivity surge followed by a stable stage. The formation of product layers acted as transport barriers, shifting C_3_A’s rapid dissolution toward diffusion-controlled regimes. Controlled particle size reduction accelerated the kinetics of dissolution and hydration compared to coarse particles. Meanwhile, annealed, low-defect C_3_A exhibited a lower heat release rate than its high-defect counterpart. Regarding ionic influences, we found that sulfates and chlorides exerted divergent effects beyond simple concentration dependence, with C_3_A exhibiting preferential reactivity toward sulfates to form AFt. By quantifying the dissolution and early hydration kinetics of the C_3_A–quartz system in aqueous solutions, this study improves our understanding of the mechanisms behind cement reactivity.

## Data Availability

The original contributions presented in this study are included in the article. Further inquiries can be directed to the corresponding authors.
